# A simple, one-step hydrothermal approach to durable and robust superparamagnetic, superhydrophobic and electromagnetic wave-absorbing wood

**DOI:** 10.1038/srep35549

**Published:** 2016-10-17

**Authors:** Hanwei Wang, Qiufang Yao, Chao Wang, Bitao Fan, Qingfeng Sun, Chunde Jin, Ye Xiong, Yipeng Chen

**Affiliations:** 1 School of Engineering, Zhejiang A & F University, Lin'an 311300, PR China; 2 Key Laboratory of Wood Science and Technology, Zhejiang Province, 311300, PR China

## Abstract

In this work, lamellar MnFe_2_O_4_ was successfully planted on a wood surface through the association of hydrogen bonds via the one-pot hydrothermal method. Simultaneously, the fluoroalkylsilane (FAS-17) on the surface of the MnFe_2_O_4_ layer formed long-chain or network macromolecules through a poly-condensation process and provided a lower surface energy on the wood surface. The MnFe_2_O_4_/wood composite (FMW) presented superior superparamagnetism, superhydrophobicity and electromagnetic wave absorption performance. The results indicated a saturation magnetization of the FMW with excellent superparamagnetism of 28.24 emu·g^−1^. The minimum value of reflection loss of the FMW reached −8.29 dB at 16.39 GHz with a thickness of 3 mm. Even after mechanical impact and exposure to corrosive liquids, the FMW still maintained a superior superhydrophobicity performance.

Currently, bamboo, wood and various other natural organic materials are usually considered good candidates as host materials for composites, and as-prepared composites can still retain their special morphology while obtaining some unique properties^1^,^2^,^3^,^4^,^5^. In particular, wood or wood-based products feature superior physical properties and a warm appearance and have excellent functions in penetrability, accessibility and reactivity, which are essentially different from other materials. However, when facing sources of environmental erosion such as acid rain and high temperatures, wood surfaces typically loses their efficacy with clouding, roughening, discolouring and checking. Therefore, potential mechanical, thermal, optical, magnetic and biological applications of wood-inorganic hybrid materials have attracted significant attention^6^,^7^,^8^. More importantly, through solutions combining inorganic particles on wood surfaces, the wood receives efficient protection from environmental erosion, which improves durability and robustness and extends the application range and service life^9^,^10^.

Oxide metal particles, such as CoFe_2_O_4_^11^,^12^,^13^, Fe_3_O_4_^14^, MnFe_2_O_4_^13^,^15^,^16^, and others^17^, are very useful inorganic materials because of their distinct size-dependent physicochemical, magnetic and other excellent properties. Among these oxide metals particles, superparamagnetic MnFe_2_O_4_ particles are a highly active area of research and can be applied to a wide range of applications, including air and water purification, ultraviolet resistance, and electromagnetic shielding. Furthermore, MnFe_2_O_4_ particles have massive hydroxyl groups on their surfaces, which can produce an effective combination with the surface of wood and may provide superior magnetic and electromagnetic wave absorption properties^18^,^19^.

Superhydrophobicity is of great interest in various fields. Superhydrophobic materials, because of their self-cleaning surfaces, biocompatibility and other excellent properties, are widely used in applications in waterproofing, anti-fouling, and bacteriostasis^20^,^21^,^22^. Often, clouding, discolouring and checking defects in wood are caused by the hydrophilicity of the wood surface. Therefore, to overcome the wood’s defects and prolong the wood’s service life, the preparation of superhydrophobic wood is necessary^23^,^24^. In general, a superhydrophobic wood surface can be prepared by two approaches: forming a layer of inorganic particles on the wood surface to create a rough structure or chemically modifying a rough surface with a low surface free energy (such as FAS-17)^25^,^26^. Most of the preparation methods for these materials are complicated and require at least two or three steps for the formation of the superhydrophobic surface. In this work, we employed a simple one-step hydrothermal method for the growth of MnFe_2_O_4_ on a wood surface, and the as-prepared MnFe_2_O_4_/wood composite exhibited superior superparamagnetism, superhydrophobic and electromagnetic wave absorption properties. In addition, the chemical and mechanical stability of superhydrophobic surfaces are very important to the performance of the material and have received broad interest. Thus, we tested the superhydrophobicity of the wood surface by sand abrasion and a test of resistance against corrosive liquids, which indicated a good chemical and mechanical stability of the wood surface when facing environmental erosion.

## Results

**Figure 1:**
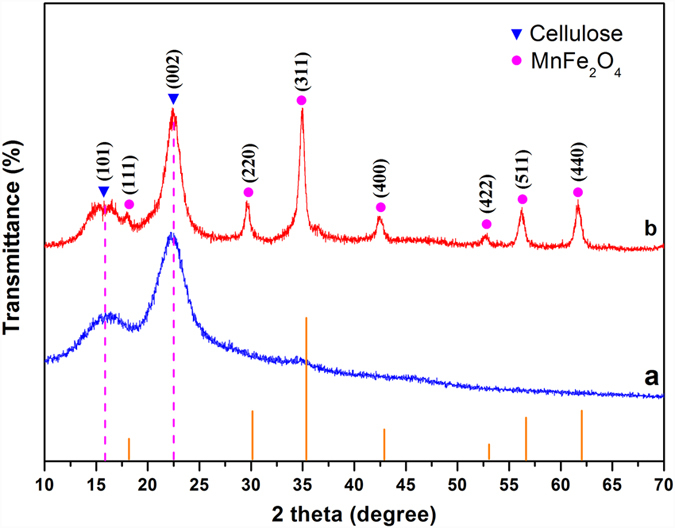
X-ray diffraction (XRD) analyses of (**a**) the wood and (**b**) the FMW.

Figure 1 shows the X-ray diffraction (XRD) patterns of the wood and the MnFe_2_O_4_/wood composite. As shown in Fig. 1a,b, strong diffraction peaks at 16.0° and 22.5° appear in the wood and the FMW spectra, which originated from the crystalline region of the cellulose in the wood^27^. Seven crystalline peaks were observed at 2θ° of 17.96°, 30.31°, 35.67°, 43.33°, 53.52°, 57.09° and 62.78° and were related to the 111, 220, 311, 400, 422, 511 and 440 crystallographic planes, corresponding to the MnFe_2_O_4_ crystals (JCPDS 73-1964) with a spinel structure^28^. Therefore, MnFe_2_O_4_ had been successfully grown on the wood surface.

**Figure 2:**
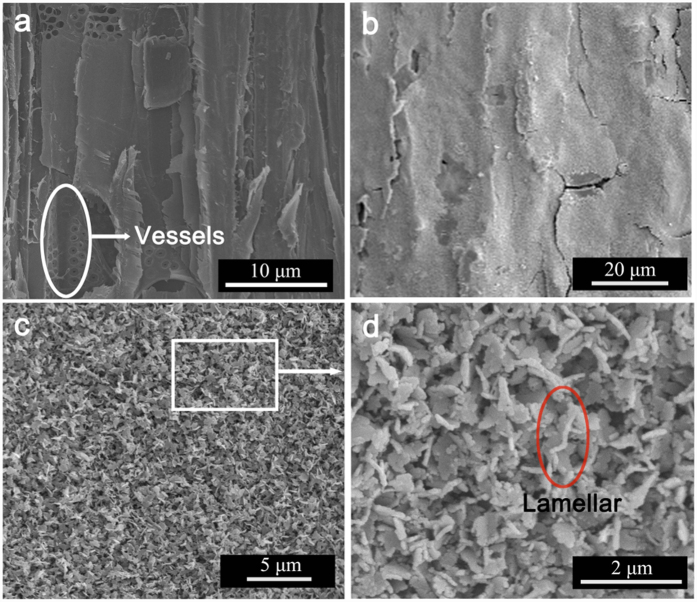
SEM micrographs of (**a**) the wood, (**b**) the surfaces of the FMW with a low-magnification and (**c,d**) local partial enlarged images of (**b**).

Figure 2 shows the surface morphologies of the wood and the FMW. As shown in Fig. 2a, the microstructure of the wood in a longitudinal section and the inner surface of the lumen and the vessels were observed on the wood surface. Figure 2b shows the surface of the FMW with a very low magnification. After the one-step hydrothermal process, the MnFe_2_O_4_ was packed tightly on the wood surface, masking the vessels and other details. The magnified SEM image of the FMW revealed that the MnFe_2_O_4_ layer was composed of a number of lamellas with different sizes (Fig. 2c,d). Therefore, these results indicate that the lamella MnFe_2_O_4_ had been successfully coated onto the wood surface.

**Figure 3:**
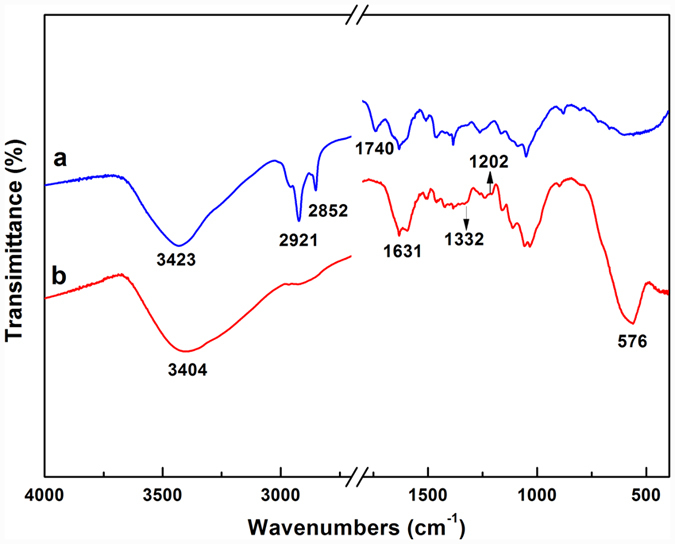
FT-IR spectra of (**a**) the wood and (**b**) the FMW.

Figure 3 shows the typical FT-IR spectra of the wood and the FMW. The peaks at 3450–3400 cm^−1^ were attributed to the **-**OH stretching absorption bands arising from the hydroxyl groups of the wood, which shifted to lower wavenumbers. This result indicates the formation of a strong interaction between the hydroxyl groups of the wood surface and the MnFe_2_O_4_ through hydrogen bonds^29^. Then, as shown in Fig. 3a, the peaks at 2921 cm^−1^, 2852 cm^−1^ and 1740 cm^−1^ were ascribed to the stretching vibrations of -CH_3_, -CH_2_ and C=O, respectively^30^. These peaks in the FMW spectra (Fig. 3b) had significantly decreased, which might have occurred by the hydrolysis of fatty acids through the alkaline hydrothermal process. More importantly, the peaks at 1202 cm^−1^ and 1332 cm^−1^ were assigned to the C-F stretching vibration, which corresponds to the FAS-17 that is incorporated into the MnFe_2_O_4_ surface^31^. In addition, a strong adsorption peak appeared at 576 cm^−1^, which was attributed to the intrinsic vibrations of the manganese ferrite (Fe-O or Mn-O)^32^. Therefore, the analysis of the FT-IR spectra of the FMW exhibited the existence of FAS-17 and hydrogen bonds between the wood surface and the MnFe_2_O_4_.

**Figure 4:**
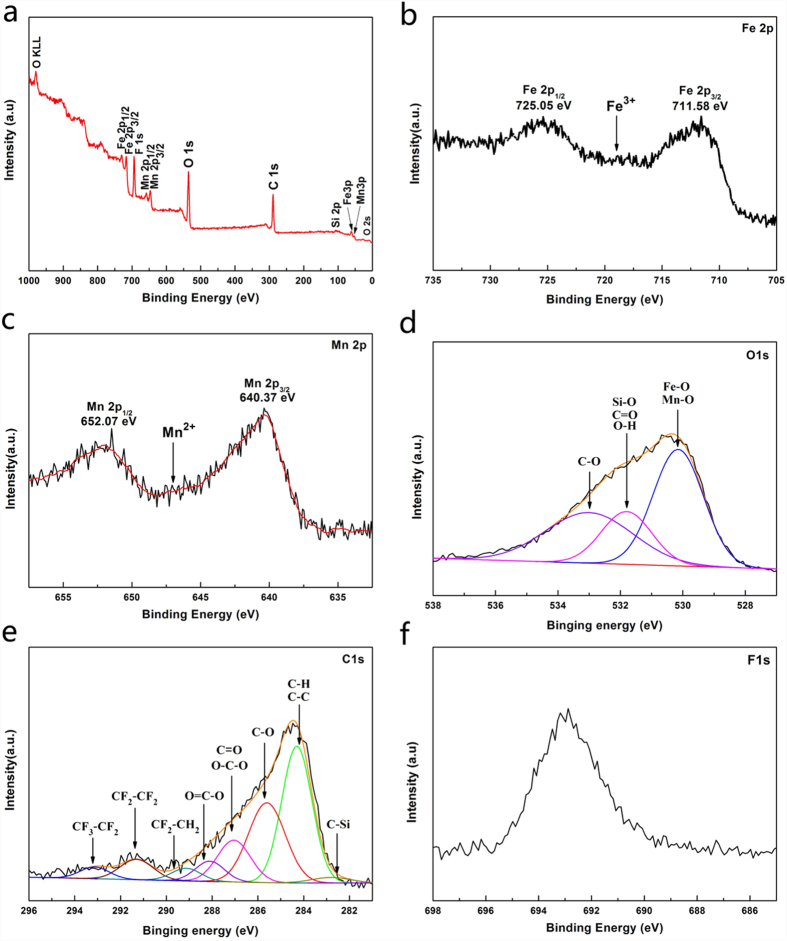
(**a**) Survey-scan XPS spectra of the FMW, (**b**) Fe2p XPS spectra of the FMW, (**c**) Mn2P XPS spectra of the FMW, (**d**) O1s XPS spectra of the FMW, (**e**) C1s XPS spectra of the FMW and (**f**) F1s XPS spectra of the FMW.

X-ray photoelectron spectroscopy (XPS) was employed as a potent technique to analyze the chemical structure characteristics and surface modification of the FMW after the hydrothermal process. The survey-scan spectra of the FMW exhibited the existence of C, O, Mn, Fe, F and Si in the FMW surface, as shown in Fig. 4a. The wood substrate provided the major source of C and O, and the MnFe_2_O_4_ growing in the wood surface provided the Fe, Mn and O. F, Si, and a portion of C and O originated from the hydrophobic layer (FAS-17) on the surface of the MnFe_2_O_4_.

The Fe 2p peak (Fig. 4b) was a doublet with a spin-orbit separation of 13.7 eV, and the Fe^3+^ cation had a well-defined structure at 8 eV after the Fe 2p_3/2_ peak (711.58 eV). However, this peak in the Fe_3_O_4_ was smeared and almost equal in proportion because of the presence of both Fe^2+^ and Fe^3+ ^33. As shown in Fig. 4c, the Mn 2p spectra consisted of spin-orbit-split 2p_3/2_ and 2p_1/2_ with a separation of 11.7 eV, which was approaching the spin-orbit separation (11.6 eV) of the MnO^34^. In addition, the satellite peak at 647.08 eV was approximately 5 eV distant from the 2p_1/2_ peak of the Mn 2p spectra, which is the typical Mn^2+^ ion behaviour^35^. Therefore, these results provided powerful evidence of the presence of Mn^2+^ and Fe^3+^ in the FMW surface.

The O 1s region (Fig. 4d) was dominated by three components centred at 530.18, 531.78 and 533.08 eV, arising from the photoelectrons ejected from the oxygen 1 s orbital. The dominant peak at 533.08 eV was attributed to the Fe-O and Mn-O from the MnFe_2_O_4_ layer^36^. The peak at 531.78 eV included a carbon-oxygen double bond (such as lignin), -OH groups (such as adsorbed water, hydroxyl groups from the wood or hydrogen bonds between the wood surface and MnFe_2_O_4_) and a Si-O bond (FAS-17 layer)^31^. Finally, the C-H bond at 533.08 eV was provided by the FAS-17 and the wood substrate.

The C 1 s spectrum (Fig. 4e) of the FMW surface could be resolved into eight components, namely, CF_3_-CF_2_ (293.13 eV), CF_2_-CF_2_ (291.30 eV), CF_2_-CH_2_ (289.13 eV), O=C-O (288.10 eV), C=O or O-C-O (287.05 eV), C-O (285.61 eV), C-H or C-C (284.30 eV) and C-Si (282.5 eV)^31^. The presence of the CF_3_-CF_2_, CF_2_-CF_2_, CF_2_-CH_2_ and C-Si in the spectrum provided powerful evidence that the hydrolytic FAS-17 had been incorporated into the MnFe_2_O_4_ surface and formed the hydrophobic layer through the self-assembly process. The other peaks originated from the wood surface (cellulose, hemicellulose and lignin), and a small amount of the hydrocarbon originated from the XPS instrument itself^37^. Figure 4f shows the high-resolution XPS F 1 s spectra of the FMW surface. The peak at 693 eV was attributed to the F 1 s from the FAS-17 layer. In summary, all of the XPS results demonstrated that the FMW sample consisted of C, O, Mn, Fe, F and Si elements with an anomic ratio of Fe:Mn at 2.35:1 and proved the presence of the MnFe_2_O_4_ and the hydrophobic layer (FAS-17 layer) on the wood surface.

**Figure 5:**
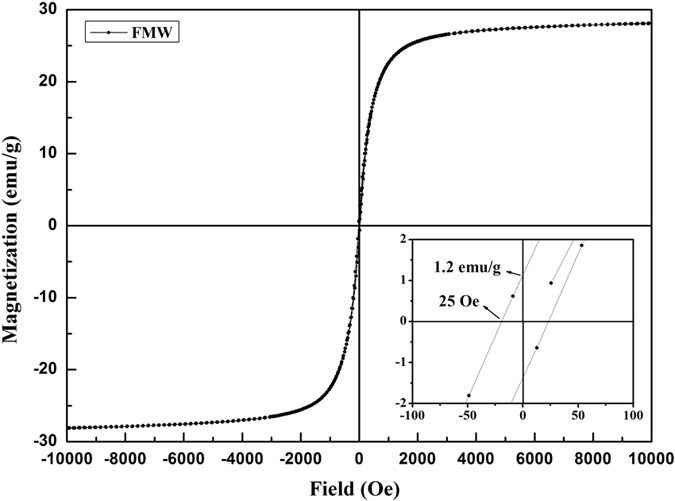
Magnetization of the FMW as a function of the applied magnetic field. The inset shows a magnification of one segment of the FMW magnetization-hysteresis curves.

Figure 5 shows the magnetization-hysteresis (M-H) curves of the FMW. The magnetic property of the MnFe_2_O_4_/wood composite was analyzed by room temperature VSM with an applied field of −10 KOe ≤ H ≤ 10 KOe. The value of the saturation magnetization (Ms) of the FMW was 28.08 emu·g^−1^. The lower right corner inset shows that the remnant magnetization (Mr) and coercivity field were 1.2 emu·g^−1^ and 25 Oe, respectively. These results show that the FMW had superior superparamagnetic properties, small hysteresis loops and low coercivity^38^. Therefore, the MnFe_2_O_4_/wood composite showed superparamagnetism via a one-pot hydrothermal process with low temperature.

The mechanism of the formation of the MnFe_2_O_4_ can be expressed by reaction Equations (1)–(3), 2, 3:


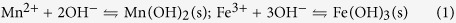



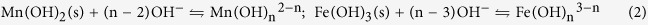



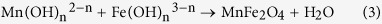


**Figure 6:**
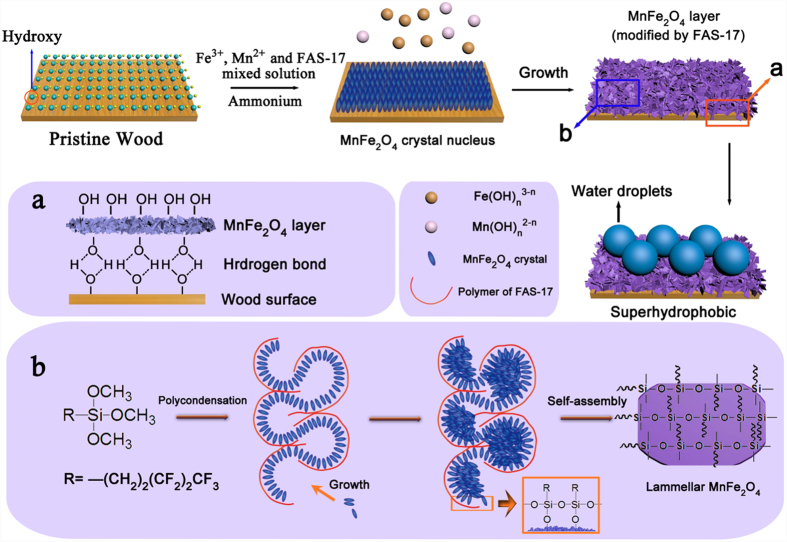
Possible schematic illustration of the preparation of the FMW. Insets (**a**,**b**) show the combination mechanism of the MnFe_2_O_4_ layer and the wood surface and the formation mechanism of the hydrophobic lamellar MnFe_2_O_4_, respectively.

According to previous results, the possible mechanism can be described as follows (Fig. 6). First, when the ammonia solution was added into the Fe^3+^, Mn^2+^ and FAS-17 mixed solution containing the wood substrate, the Fe^3+^ and Mn^2+^ ions transformed into Fe and Mn hydroxides, respectively. Then, part of the Fe and Mn hydroxides dissolved in the mixed solution because of the presence of high concentrations of the ammonia and converted to the Mn(OH)_n_^2−n^ and Fe(OH)_n_^3−n^ with massive hydroxyl groups. Second, the Mn(OH)_n_^2−n^ and Fe(OH)_n_^3−n^, as a growth unit, formed the MnFe_2_O_4_ crystal nucleus on the wood surface by the dehydration reaction. Then, the MnFe_2_O_4_ crystal grew with the deposition of the MnFe_2_O_4_ nucleus, which eventually formed a more stable MnFe_2_O_4_/wood composite. More importantly, inset a shows the combination mechanism of the MnFe_2_O_4_ and the wood surface by the hydrogen bonds. As shown in inset b, the Si-OCH_3_ groups in the FAS-17 molecules participate in a hydrolysis reaction, which caused the elimination of CH_3_OH and the formation of the Si-OH groups. Subsequently, after hydrolysis, the FAS-17 molecules on the MnFe_2_O_4_ crystal nucleus surface formed long-chain or network macromolecules by a polycondensation reaction. Then, the MnFe_2_O_4_ crystal grew in the constraint of the fluoroalkylsilane macromolecule structure and, ultimately, formed the lamellar MnFe_2_O_4_ on the wood surface. Finally, when water was dropped onto the wood surface, the FMW displayed a superior superhydrophobic property.

**Figure 7:**
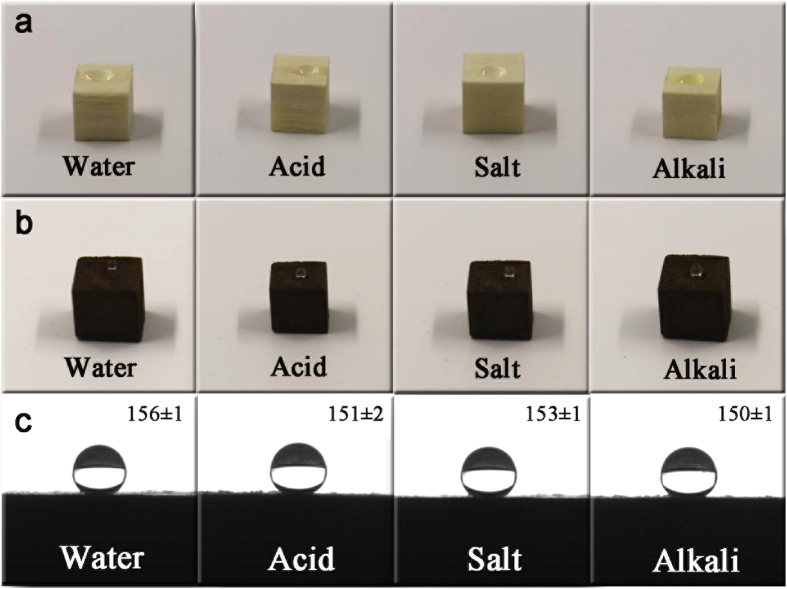
Optical images of water, HCl solution (pH 1), NaCl solution (pH 7) and NaOH solution (pH 14) droplets on the surfaces of (**a**) wood and (**b**) FMW, and (**c**) the contact angle values and profiles of the FMW.

To provide a more visible demonstration of the superhydrophobic performance, four types of corrosive liquids were placed on the surface of the wood and FMW, including water, acid, salt, and alkali droplets. Figure 7a shows that all of the liquid profiles on the wood surface were approximately zero, which proved the hydrophilicity of the pristine wood. In contrast, the surface of the FMW was highly repellent to the water and other liquids, and the droplets exhibited spherical shapes on the sample surface (Fig. 7b). Figure 7b shows the contact angle values and profiles of several typical liquids. The CA value of the water droplet on the FMW surface was approximately 156°, and all of the CA values of the acid, salt, and alkali droplets on the sample surface were over 150° because the high surface concentration of -CF_3_ and -CF_2_ from the FAS-17 layer provided a low surface energy on the FMW surface, which is conducive for superior superhydrophobic performance.

**Figure 8:**
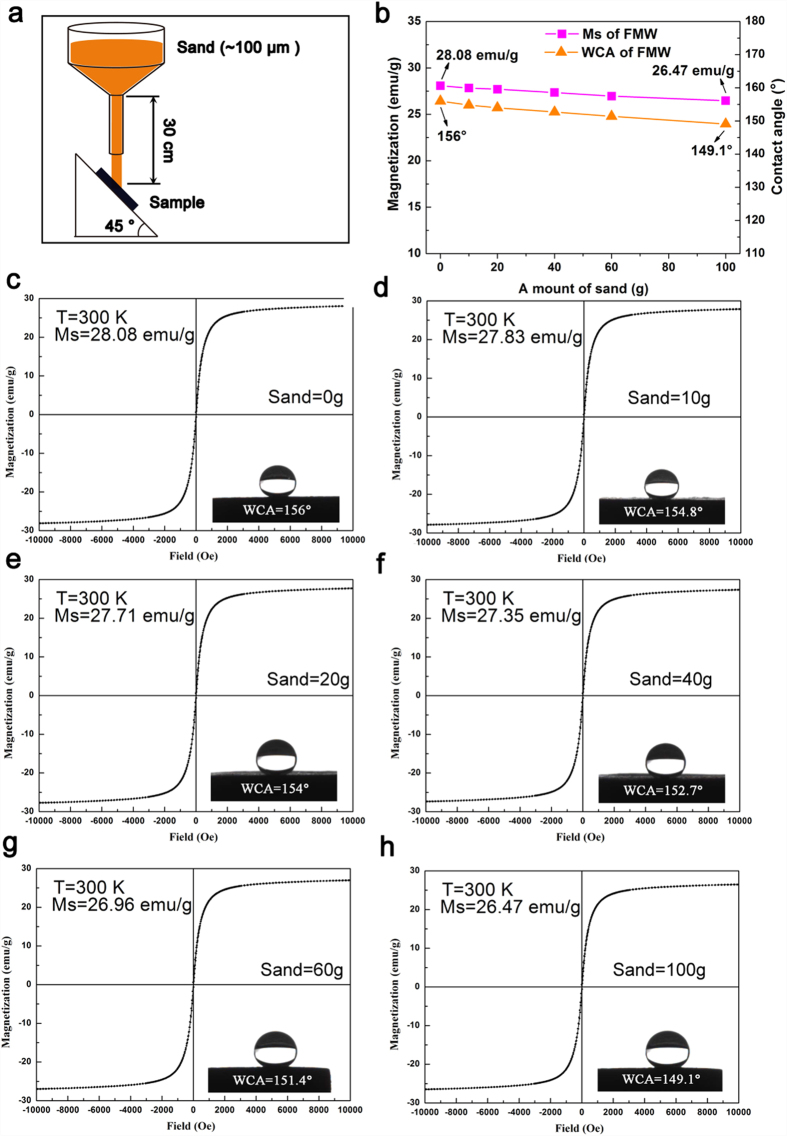
Schematic description of the experimental set-up for (**a**) the sand abrasion experiment of the FMW. (**b**) The trend graph of Ms and WCA of the FMW as a function of the amount of sand dropped onto the surface. (**c–h**) The values of saturation magnetization (Ms) and WCAs with its corresponding curves or image varied with the mass of sand dropped onto the surface.

The superhydrophobic property of the FMW was further evaluated by a sand abrasion experiment. In this test, sand grains (10 g) with an average diameter of approximately 100 μm were flowed to the FMW surface from a height of 30 cm, and the sand grains repeatedly impacted the FMW surface 10 times (100 g of sand). As shown in Fig. 8b, it is intuitive to show that the values of the contact angles of water (WCA) and the saturation magnetization (Ms) of the FMW gradually decreased with increases in the experiment frequency. The WCA images and Ms curves of the FMW with its values were recorded at 0 g, 10 g, 20 g, 40 g, 60 g and 100 g, shown in Fig. 8c–h, respectively. Before the experiment, the WCA of the FMW was 156°. After 10 cycles of the sands abrasion test, the WCA of the FMW was 149.1°. In addition, the value of the Ms of the FMW had only decreased by 1.61 emu g^−1^, indicating its stable performance for superhydrophobicity and superparamagnetism. Therefore, these results indicate that the samples have excellent durability magnetism and mechanical stability when confronted with external abrasion, and the FMW might have great potential in a wide range of applications.

**Figure 9:**
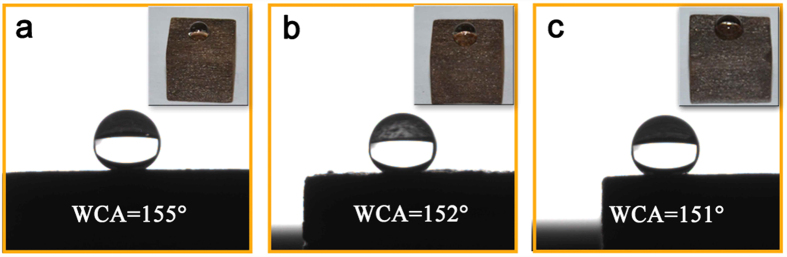
The water contact angle profiles and values of the samples with an immersion time in solutions of (**a**) NaCl (0.1 mol/L), (**b**) HCl (pH 1) and (**c**) NaOH (pH 14) at 50 °C for 4 h.

To evaluate the durability of the superhydrophobic wood surface for mild acids and strong bases, the as-prepared FMW samples were placed into a NaCl solution of 0.1 mol/L (Fig. 9a) HCl solution with a pH of 1 (Fig. 9b) and a NaOH solution with a pH of 14 (Fig. 9c) for 4 h at 50 °C. In contrast to the baseline condition, the WCA of the FMW surface displayed only a small change, and the water contact angles were larger than 150°. These results showed that all of the samples maintained their superhydrophobic properties after the acid and base corrosion tests and had good durability towards acidic (152°) and basic (151°) liquids. In addition, the salt solution corrosion test demonstrated that Cl and Na ions had almost no effect on the superhydrophobic performance under the same conditions (Fig. 9a). Figure 9b,c show that the acid resistance of the FMW was appreciably stronger than the base resistance. Therefore, this superhydrophobic MnFe_2_O_4_/wood composite might have applications in outdoor building materials to protect wood substrates from acid rain erosion by the environment.

**Figure 10:**
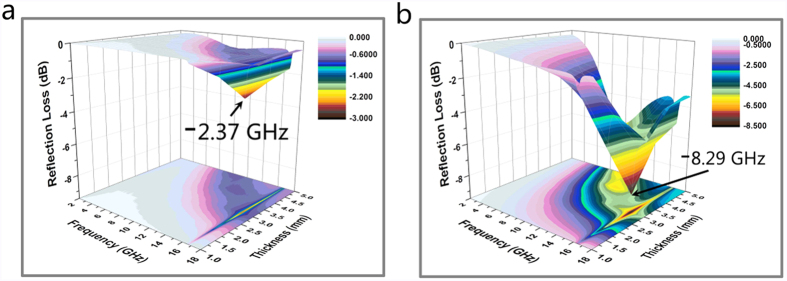
Frequency dependence of the reflection loss for (**a**) the wood and (**b**) the FMW by three-dimensional and colour-filling patterns in the frequency range of 2–18 GHz.

To investigate the electromagnetic absorption property of the wood and the FMW, the reflection loss (RL) values were calculated by the transmission line theory^39^.


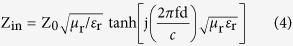



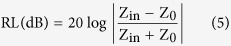


where Z_in_ is the input impedance of the samples, Z_0_ is the impedance of air, *ε*_r_ is the complex permittivity, *μ*_r_ is the complex permeability, d is the thickness of the samples, c is the velocity of light and f is the microwave frequency. The values of the reflection loss (RL) of the wood and the FMW are exhibited by three-dimensional and colour-filling pattern images in the frequency range of 2–18 GHz in Fig. 10. Figure 10a,b show that both the wood and FMW had a minimum value at the thickness of 3 mm, and the corresponding values of the reflection loss were −2.37 dB at 16.64 GHz and −8.29 dB at 16.39 GHz, respectively. Since the magnetic loss of MnFe_2_O_4_ after the lamellate MnFe_2_O_4_ formed on the wood surface, the absorption bandwidth of the FMW improved significantly compared with the wood. These results indicate that the electromagnetic absorption property of the MnFe_2_O_4_/wood composite was improved via a simple one-step hydrothermal method, which might provide potential applications in electromagnetic wave-absorbing materials and electromagnetic shielding.

## Conclusions

In summary, we developed a simple one-step hydrothermal method to fabricate a superparamagnetic MnFe_2_O_4_/wood composite with good chemical and mechanical stability of its superhydrophobic and electromagnetic wave absorption properties. The reaction involved hydrogen bond assembly and a polycondensation process. The hydrogen bond assembly appeared in the formation process of the lamellar MnFe_2_O_4_ layer, causing the tight adhesion of the lamellar MnFe_2_O_4_ onto the wood surface. The fluoroalkylsilane macromolecules on the MnFe_2_O_4_ layer surface via the polycondensation process provided a low surface energy to achieve superhydrophobicity. After a one-step hydrothermal reaction, the value of the saturation magnetization of the FMW reached 28.08 emu·g^−1^ and presented excellent superparamagnetism properties. The value of the water contact angles of FMW reached 156°, and the FMW maintained its superhydrophobic properties even after sand abrasion and exposure to corrosive liquids. In addition, the value of the minimum reflection loss of the FMW reached −8.29 dB at 16.39 GHz with a thickness of 3 mm, and the absorption bandwidth improved. These findings suggest that the MnFe_2_O_4_/wood composite has potential applications in electromagnetic wave-absorbing materials, electromagnetic shielding and outdoor building materials.

## Methods

### Materials

All chemicals were supplied by Shanghai Boyle Chemical Co. Ltd (Shanghai. China). and used without further purification. The wood slices were cut to dimensions of 10 mm (length) × 10 mm (width) × 10 mm (height), and then the slices were ultrasonically rinsed with deionized water for 30 minutes and dried at 80 °C in a vacuum.

### One-pot hydrothermal synthesis of FMW

In a typical synthesis, FeCl_3_∙6H_2_O (3.24 g) and MnCl_2_∙4H_2_O (0.94 g) in a stoichiometric ratio of 2:1 were dissolved in 80 mL of a deionized water solution with the wood samples under magnetic stirring for 2 h at room temperature. During the stirring process, FAS-17 (1 mL) and the proper amount of ammonia (3 mL) were added dropwise into the solution. Then, the obtained homogeneous mixture was transferred into a 100 mL Teflon-lined stainless autoclave, and this vessel was sealed and heated to 120 °C for 8 hours. Subsequently, the autoclave was left to cool to room temperature. Finally, the prepared magnetic wood samples were removed from the solution, ultrasonically rinsed with deionized water for 30 minutes, and dried at 45 °C for over 24 hours in vacuum.

### Characterizations

The surface morphologies of the samples were characterized by scanning electron microscopy (SEM, FEI, Quanta 200). Crystalline structures of the samples were identified by the X-ray diffraction technique (XRD, Rigaku, D/MAX 2200), operated with Cu Kα radiation (λ = 1.5418 Å) at a scan rate (2θ) of 4° min^−1^, an accelerated voltage of 40 kV and the applied current of 30 mA ranging from 10° to 70°. Changes in the chemical groups were recorded via Fourier transform infrared spectroscopy (FT-IR, Magna-IR 560, Nicolet). XPS analysis was characterized by an X-ray photoelectron spectrometer (ESCALAB 250 XI, ThermoFisher Co.). The magnetic properties of the composites were measured by a vibrating sample magnetometer (VSM, LakeShore, Model 7404, USA) at 300 K. The contact angle analyzer (CAA, JC2000C, Powereach Co., China) at ambient temperature with a droplet volume of 5 μL was employed to measure the contact angles (CAs) of the samples. An average of the five measurements taken at different positions on each sample was applied to calculate the final CA value. The sand abrasion test evaluated the mechanical resistance of the MnFe_2_O_4_/wood composite. Sand (10 g in each test with an average particle size of 100 μm) was dropped from a 30 cm height onto the fabricated films, at a tilt angle of 45° with respect to the axis vertical to the bottom surface of the experimental set-up. The microwave parameters of the samples were measured at 2–18 GHz with an AV3618 network analyzer. The reflection losses (dB) of the composites were calculated according to the transmission line theory, using the measured data of relative complex permeability and permittivity.

## Additional Information

**How to cite this article**: Wang, H. *et al.* A simple, one-step hydrothermal approach to durable and robust superparamagnetic, superhydrophobic and electromagnetic wave-absorbing wood. *Sci. Rep.* **6**, 35549; doi: 10.1038/srep35549 (2016).
